# Supplementary material for: The association of fear of movement and postural sway in people with low back pain
Source: Front Psychol. 2022 Nov 18;13:1006034. doi: 10.3389/fpsyg.2022.1006034 (PMC9716132; doi:10.3389/fpsyg.2022.1006034)
Supplement: Supplementary file 1 [file Data_Sheet_1.pdf]

Supplementary Material 1

Supplementary Table 1. Spearman correlation coefficients and corresponding P-Values for fear and displacement on the sagittal and frontal plane during both assessments.

	Displacement Sagittal				Displacement Frontal			
	Assessment T1		Assessment T2		Assessment T1		Assessment T2	
	r	P	r	P	r	P	r	P
TSK-11	-.00	.997	.13	.576	.20	.389	.31	.173
Fear Sagittal	-.26	.273	.01	.962	.19	.427	.25	.267
Fear Frontal	.19	.412	.34	.128	.49	.029	.54	.011
Relative Directional Fear	-.35	.130	-.48	.027	-.17	.468	-.37	.101

Supplementary Table 2. Spearman correlation coefficients and corresponding p-values for fear and velocity on the sagittal and frontal plane during both assessments.

	Velocity Sagittal				Velocity Frontal			
	Assessment T1		Assessment T2		Assessment T1		Assessment T2	
	r	P	r	P	r	P	r	P
TSK-11	.39	.090	.19	.400	.35	.133	.43	.049
Fear Sagittal	.30	.206	.37	.102	.23	.332	.44	.049
Fear Frontal	.13	.585	.40	.072	.47	.039	.61	.003
Relative Directional Fear	-.01	.952	-.27	.231	-.31	.183	-.33	.149
