# Supplementary material for: The association of fear of movement and postural sway in people with low back pain
Source: Front Psychol. 2022 Nov 18;13:1006034. doi: 10.3389/fpsyg.2022.1006034 (PMC9716132; doi:10.3389/fpsyg.2022.1006034)
Supplement: Supplementary file 2 [file Data_Sheet_2.pdf]

## *Supplementary Material 2*

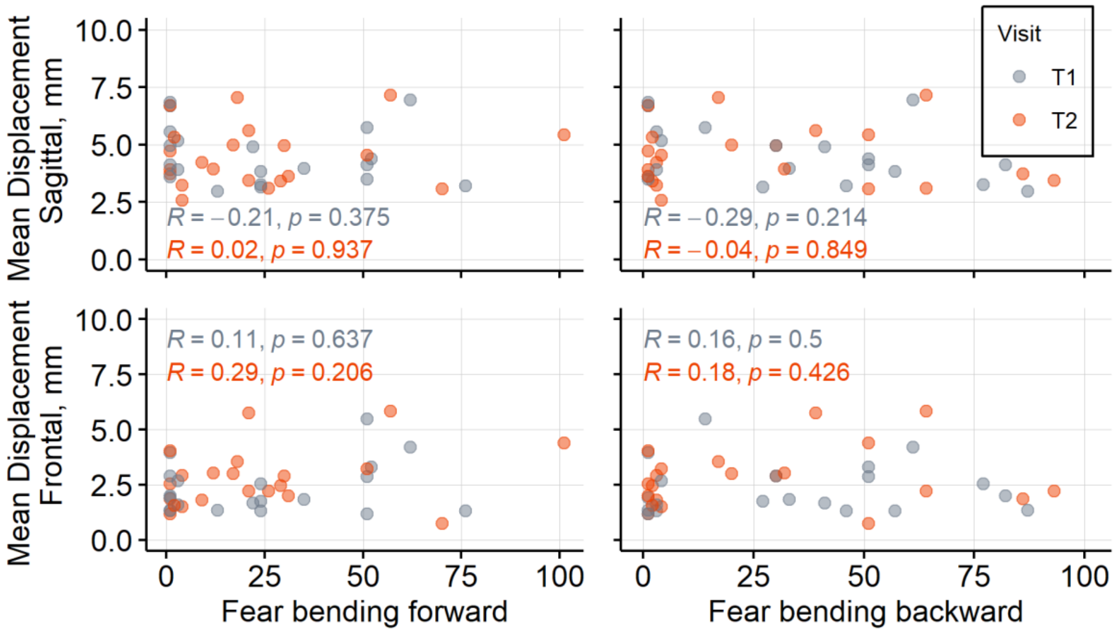


**Supplementary Figure 1**. Postural sway displacement and fear of bending forward and fear of bending backward. R values are spearman correlation coefficients.

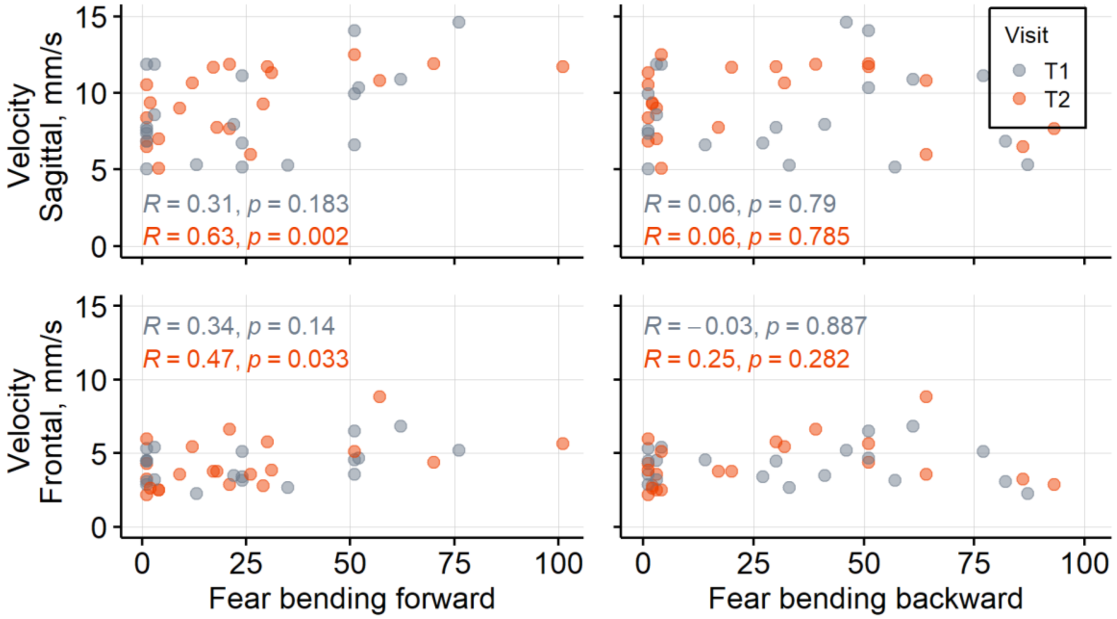


**Supplementary Figure 2**. Postural sway velocity and fear of bending forward and fear of bending backward. R values are spearman correlation coefficients.
